# 3D-Printed Scaffolds and Biomaterials: Review of Alveolar Bone Augmentation and Periodontal Regeneration Applications

**DOI:** 10.1155/2016/1239842

**Published:** 2016-06-05

**Authors:** Farah Asa'ad, Giorgio Pagni, Sophia P. Pilipchuk, Aldo Bruno Giannì, William V. Giannobile, Giulio Rasperini

**Affiliations:** ^1^Department of Biomedical, Surgical and Dental Sciences, Foundation IRCCS Ca' Granda Polyclinic, University of Milan, Milan, Italy; ^2^Department of Periodontics and Oral Medicine, School of Dentistry, University of Michigan, Ann Arbor, MI, USA; ^3^Department of Biomedical Engineering, College of Engineering, University of Michigan, Ann Arbor, MI, USA

## Abstract

To ensure a successful dental implant therapy, the presence of adequate vertical and horizontal alveolar bone is fundamental. However, an insufficient amount of alveolar ridge in both dimensions is often encountered in dental practice due to the consequences of oral diseases and tooth loss. Although postextraction socket preservation has been adopted to lessen the need for such invasive approaches, it utilizes bone grafting materials, which have limitations that could negatively affect the quality of bone formation. To overcome the drawbacks of routinely employed grafting materials, bone graft substitutes such as 3D scaffolds have been recently investigated in the dental field. In this review, we highlight different biomaterials suitable for 3D scaffold fabrication, with a focus on “3D-printed” ones as bone graft substitutes that might be convenient for various applications related to implant therapy. We also briefly discuss their possible adoption for periodontal regeneration.

## 1. Introduction

Placement of endosseous implants has revolutionized modern dentistry, with a constantly increasing number of patients seeking replacement of lost teeth with this modality of treatment.

Since the overall success of dental implant therapy depends on the presence of adequate bone volume at implant sites [1], sufficient vertical and horizontal amounts of alveolar ridge prior to dental implant placement are essential especially in the anterior maxilla, which is a highly demanding aesthetic region.

Bone augmentation can be carried out using different techniques: bone blocks or guided bone regeneration (GBR) is mainly applied for horizontal grafting [2]. Vertical bone augmentation employs more challenging and technique-sensitive methods (vertical GBR, onlay grafting, inlay grafting, and distraction osteogenesis [3, 4]) and is frequently associated with high complication rates such as soft tissue dehiscence and subsequent exposure of bone grafts to the oral cavity [5].

In an attempt to overcome the obstacles related to vertical bone augmentation, short dental implants have been suggested as an alternative in the atrophic areas [6]. Despite being an acceptable option in the posterior areas of both jaws, bone grafting is still obligatory in anterior regions with severe bone resorption to achieve final satisfactory aesthetic results.

Bone grafts serve as filling materials with alternating properties of space maintenance, blood clot stabilization, and scaffolding [7], by providing a temporary template to support migration of cells from the periphery of the grafted area [8]. Bone grafting materials are divided into autografts, allografts, xenografts, and alloplasts, each with its own set of advantages and disadvantages [9]. As a result, researchers are constantly working on exploring new bone graft substitutes with more predictable regenerative outcomes and minimal complications. To this end, tissue engineering has become more commonly used for oral bone grafting procedures.

The specific field of tissue engineering that mainly focuses on enhancing bone regeneration and repair by creating substitutes to traditional bone grafting materials is referred to as bone tissue engineering (BTE) [10] which started about three decades ago and has been witnessing a tremendous growth ever since [11]. This could be ascribed to the high regenerative potential of bone in comparison to other tissues in the body, thus serving as a paradigm for general principles in tissue engineering [12]. A classic BTE paradigm includes the following three key components: biomaterials to provide a scaffold for new tissue growth, cells, and signaling molecules [11, 13].

Within this model, scaffolds can be either acellular or cellular upon implantation. In the former, the overall architecture and geometry promote the recruitment of local stem cell and or/osteoprogenitor cells [14], which could be possible with “smart” cues and attachment motifs within the scaffold architecture. On the other hand, the latter strategy involves implantation of a scaffold combined with stem cell and or/osteoprogenitor cells [14], which can be incorporated by two methods: (i) cell seeding into a “prefabricated” scaffold, a commonly applied tissue engineering strategy, and (ii) cell encapsulation during scaffold fabrication made of hydrogel polymer matrix [15], based on the immobilisation of cells within a semipermeable membrane. This technique protects cells from the immune system [16] and permits uniform cell distribution within the construct [17].

In this narrative review, based on orthopaedic and dental studies available on PubMed, MEDLINE, and Google Scholar, we focus on the first key component of the tissue engineering paradigm for applications in alveolar bone and periodontal tissue regeneration, since scaffolds are considered the key players in successful tissue formation [14]. Biomolecules and cellular elements of the paradigm for this specific application are discussed elsewhere [18].

## 2. Properties of 3D Scaffolds for Applications in Alveolar Bone and Periodontal Tissue Regeneration

Although conventional bone grafting materials serve the role of a supporting matrix, they have several disadvantages: allografts, xenografts, and alloplasts are brittle, poorly processable into porous forms, and are unable to generate structures tailored to the specific needs of patients. Likewise, they are unable to maintain the desired generated tissue volume under mechanical forces, hindering their ability to provide a proper template for effective cell interaction [8]. Although autografts may have the ability to withstand mechanical forces, they are difficult to shape and conform to a bony defect [19], which is of a significant concern in the craniofacial region.

BTE has opened new doors for regeneration through the introduction of scaffolds which possess three-dimensional (3D) architecture that closely mimics native extracellular matrix (ECM). Such arrangements eventually enhance cell adhesion, proliferation, differentiation, and overall tissue regeneration [20]. As a matter of fact, scaffold properties are influenced by the used biomaterials and must be specific for the application while in harmony with the native environment to ensure that the defect area is replaced with a healthy, functional tissue matching the original one, without reparative scar formation [21].

In general, scaffolds must exhibit an adequate degree of hydrophilicity [22, 23], roughness [24], and specific surface topography; a topographic landscape on micro- and submicrometer scales must be developed to replicate the natural process of bone regeneration [25]. Nanotopography increases the overall surface area, surface-to-volume ratio, and surface roughness [26], which enhance the adhesion between osteoblasts and the underlying scaffold surfaces [27]. As for microscale features, they facilitate cell penetration, vascularization, and diffusion of nutrients [28] and offer better spatial organization for cell growth and ECM production [29]. Development of a multiscale scaffold has been emphasized in periodontal tissue regeneration [30].

Other important design characteristics are overall porosity, pore size, and interconnectivity. As human cancellous bone demonstrates a total porosity between 30% and 90%, any construct enclosing voids within this range is considered suitable for bone regeneration [31]. However, extremely high porosity can jeopardize the overall mechanical stability of a scaffold by reducing its overall compressive strength [32]. For alveolar bone regeneration applications, an overall porosity of 70% has been applied in preclinical and clinical studies [33–35]. Regarding pore diameter, a range between 150 *μ*m and 500 *μ*m facilitates vascularization and penetration of new tissues [36] without compromising the mechanical strength of the scaffold [11] or cell infiltration into inner surface areas [37]. These consequential events are also dictated by the presence of an interconnected pore network, which is essential for cell growth into the interior of the scaffold to prevent core necrosis [38].

To achieve success in bone regeneration, the template should demonstrate mechanical strength close to native tissues to support target cells, the surrounding tissues, and newly formed ones, mainly in load-bearing areas, until full tissue formation is achieved [39, 40]. In order to maintain this process, degradation rate of a scaffold should be in concordance with the remodeling processes of the target tissue [41]. For dentoalveolar reconstruction, degradation within 5-6 months is considered appropriate [42].

In addition, as implanted scaffolds should be biocompatible and bioactive, the utilized biomaterials should not elicit any inflammatory or cytotoxic reactions [43] and must evoke a specific biological response at the interface of the material, which results in the formation of a bond with the tissues [44].

**Figure 1:**
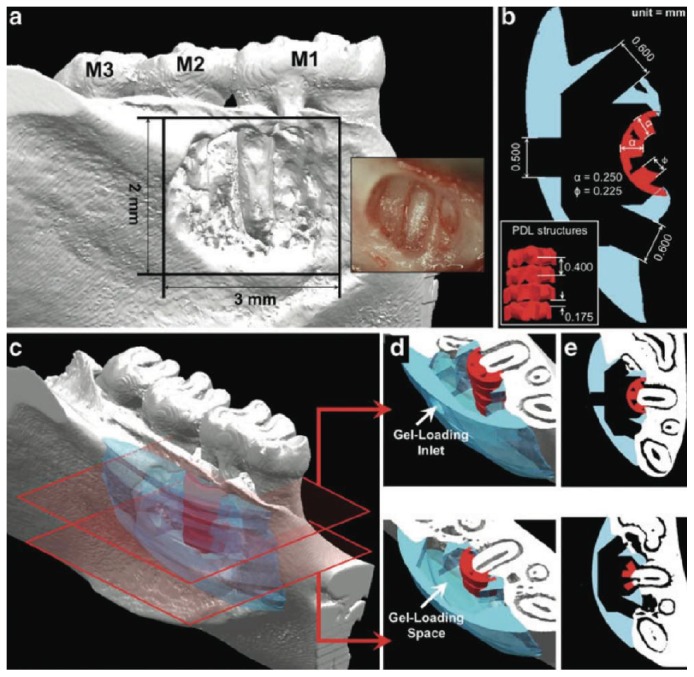
Multiphasic scaffold aimed at multiple tissue regeneration (periodontal ligament, cementum, and alveolar bone). Courtesy of Park et al., 2012 [46].

Although the previously presented features constitute the basics in scaffold designing for bone regeneration, it must be noted that the design and balance between biomaterials and scaffolds are a complex and interdisciplinary matter. Furthermore, this aspect can become more complicated when alveolar bone regeneration is attempted along with cementum and periodontal ligament tissues. In this scenario, spatial organization is necessary by utilizing a multiphasic scaffold, which encloses variable architectural and chemical composition to closely capture the structural organization of native tissue and/or its cellular and biochemical composition [45]. Therefore, “compartmentalization” is essential for controlling the spatiotemporal events resulting in effective regeneration of the periodontal complex [45] which could prevent tooth ankylosis. This can be achieved by ensuring compartmentalized formation of bone and functionally oriented periodontal ligament fibers (PDL) that are integrated over time [45].  Figure 1 illustrates a multiphasic scaffold with channel-like “*fiber-guiding architecture*” of the PDL compartment displaying a thickness of 0.250 mm to mimic the width of an adult periodontal ligament space [46].

## 3. Applied Biomaterials Used in the Fabrication of 3D Scaffolds for Alveolar Bone Regeneration

As biomaterials strongly influence the overall properties of a scaffold, it is important to comprehend their individual characteristics to allow for appropriate selection in specific applications taking into consideration the notion that biomaterials differ in their cellular affinity [47], which influences adhesion, proliferation, and the overall regeneration outcome. As cell adhesion is mediated via integrins, such differences between biomaterials can be further explored. Below, we present biomaterials that can be mainly applied in alveolar bone regeneration and are compatible with new scaffold fabrication techniques.

### 3.1. Biodegradable Natural Polymers

Natural polymers, which include proteins and polysaccharides, are the first biomaterials to be recruited in different clinical applications because of their high biocompatibility, good cell recognition, enhanced cellular interactions in the surrounding environment [48], and hydrophilicity [49]. Due to these properties, they have been thoroughly investigated as hydrogels in the earliest work of cell encapsulation in tissue engineering, demonstrating successful results [50–54].

*Collagen* is one of the most widely expressed proteins in the human body, providing strength and structural stability to many tissues from skin to bone [55]. Being the major organic component of the ECM in native bone makes collagen an attractive biomaterial for BTE applications [56]. It is well documented that collagen matrices promote cell adhesion, proliferation, and osteogenic differentiation of bone marrow stromal cells,* in vitro* [55]. Similarly, the denatured form of collagen termed* gelatin* [57] enhances osteoblast adhesion, migration, and mineralization as it contains several biological and functional groups that promote such activities [58].

Regarding polysaccharides,* chitosan* is a popular biomaterial in bone tissue engineering due to its appealing characteristics; it displays antibacterial and antifungal activities, rapid blood clot formation, and analgesic properties [59], all of which render chitosan useful in wound healing acceleration that would minimize the risk of scaffold contamination and postoperative infections, thus preventing eventual exposure and failure of the scaffold.

For the same applications,* alginate* is another commonly investigated polysaccharide. It is highly processable into different scaffold types, which encourages its employment in BTE and regenerative medicine [60], and has been the most studied biomaterial for encapsulation of living cells [16]. Interestingly, alginate and chitosan do not exist within the human body, but they display structural similarities to glycosaminoglycans (GAGs) found in the ECM of human tissues such as bone [61], making them attractive candidates for applications in tissue regeneration.

Despite their good biological properties, the previously mentioned natural polymers lack* bioactivity* [62], which is the key factor in promoting hard tissue formation. They also share weak mechanical characteristics and somewhat rapid degradation rate [60, 63, 64] through enzymatic reaction [65].

To overcome such undesired properties, scaffolds based on natural polymers are usually combined with bioactive materials (e.g., bioceramics) or mechanically strong ones (e.g., synthetic polymers or metals), depending on the area of application (e.g., load-bearing or not). Interestingly, although bioceramics are mechanically weak as well, they tend to increase the overall compressive strength of natural polymer based scaffolds [66].

### 3.2. Biodegradable Synthetic Polymers

Biodegradable synthetic polymers have generated interest in BTE because of their relatively low cost and ability to be produced in large quantities with long shelf life in comparison to their natural counterparts [37]. The most investigated biomaterials of this group are* aliphatic polyesters* which include polycaprolactone (PCL), polylactic acid (PLA), polyglycolic acid (PGA), and their copolymer poly(lactic-co-glycolic) acid (PLGA).

*Polycaprolactone* (PCL) is the most popular aliphatic polyester in medical applications; it has been used in medical devices for the last 30 years [35] and has been investigated in craniofacial repair [67]. PCL is an excellent candidate for BTE applications due to its biocompatibility [68], suitability for various scaffold fabrication techniques [69], remarkably slow degradation rate, and mechanical stability [40]. It is suggested that the latter two traits might allow for a better maintenance of generated bone volume and its contour over time. However, PCL is hydrophobic in nature [70] which is also responsible for the inferior cell affinity and poor cellular responses and interactions to the surface [71]. Similar to PCL,* polylactic acid* (PLA) and* poly(lactic-co-glycolic acid)* (PLGA) are hydrophobic while* polyglycolic acid* (PGA) is hydrophilic, keeping in mind that these polymers still have higher rates of degradation in comparison to PCL [72]. But, in general, aliphatic polyesters display a slow degradation rate in correlation to natural polymers and bioceramics [73]. Synthetic polymers degrade by hydrolysis [65] which can be in the form of bulk degradation or surface erosion [74, 75]. Most of the available polyesters degrade by the former mechanism [76] characterized by hydrolysis within the interior part of the biomaterial, resulting in an empty shell formation, while the size is maintained for a considerable amount of time [77]. This feature is considered appealing for scaffold utilization as a bone graft substitute and less suitable for drug-delivery purposes. Still, aliphatic polyesters release acidic byproducts upon degradation, which can result in tissue necrosis and subsequent scaffold failure with chronic exposure [11]. Therefore, they are usually combined with bioceramics that enhance the bioactivity of a construct and tend to neutralize the acidic byproducts by elevating the overall pH value for the scaffold [78] to maintain tissue health. Counteracting acidic byproducts and overall pH buffering can also be achieved when polyesters are combined with metals [79]. Despite the acidic byproducts and the lack of* bioactivity*, aliphatic polyesters are moldable for fabrication into the required shapes and have good mechanical properties [80, 81].

### 3.3. Bioceramics

Bioceramics are inorganic biomaterials constituting different categories, among which are calcium phosphate bioceramics and bioactive glass with very well-documented applications as bone fillers in the dental field [82]. Calcium phosphate bioceramics enclose hydroxyapatite (HAp), tricalcium phosphate (*α*-TCP and*β*-TCP), and biphasic calcium phosphate (BCP), all of which can also be in the form of injectable cement materials (pastes) that are moldable and easy to handle and harden when left in situ. Moldable calcium phosphate materials allow for intimate adaptation to complex defects, which is difficult to accomplish with conventional bone grafting materials [83].

Bioceramics are attracting more attention in bone reconstruction due to their unlimited availability, bioactivity, excellent biocompatibility, hydrophilicity, similarity to native bone inorganic components, osteoconductivity [29], and reported potential osteoinductivity [84], which is the ability to induce ectopic bone formation by instructing the surrounding* in vivo* environment to do so [85]. This potential activity can be attributed either to the surface of bioceramics which absorbs and exhibits osteoinductive factors or to the gradual release of calcium and phosphate ions into the surrounding environment, subsequently stimulating the differentiation of osteoprogenitor cells into osteoblasts. Still, both theories are yet to be confirmed [86]. The importance of incorporating calcium phosphates in 3D scaffolds for alveolar bone regeneration has already been demonstrated in the literature [34].

The most investigated calcium phosphate ceramic in BTE is* hydroxyapatite* (HAp) because it shares the same chemical composition of native bone minerals, which positively influences adhesion and proliferation of osteoblasts [87]. Despite this important feature, HAp takes a long time to degrade when in the “crystalline form”* in vivo*, causing the remaining particles to impede complete bone formation and increase the risk for infection and exposure in oral and maxillofacial regions [88]. Consequently, applications of crystalline HAp are being eventually substituted by amorphous hydroxyapatite, which has a faster degradation rate [89]. Modification of HAp degradation rate can also be achieved by its combination with other biomaterials of faster kinetics, such as natural polymers [90].

The second most widely studied calcium phosphate ceramic is *β-tricalcium phosphate* (*β*-TCP), because of its ability to form a strong bone-calcium phosphate bond [84] and its faster degradation rate [9]. Interestingly, when tricalcium phosphate is combined with HAp, a mixture termed biphasic calcium phosphate (BCP) is produced [91]. In comparison to other calcium phosphate ceramics, BCP has significant advantages in terms of controlled bioactivity, stability, while promoting bone ingrowth especially in large bone defects [92], and controllable degradation rate [93] as BCP has a higher degradation rate than HAp, yet slower than that of *β*-TCP [94].

Another biomaterial that belongs to bioceramics and is investigated in BTE is* bioactive glass* (BG), which is a silicon oxide with substituted calcium [18]. When exposed to body fluids, a layer of calcium phosphate forms on the surface of bioactive glass, which chemically binds to bone [95]. The specific type of bioglass used as a synthetic graft in intraoral applications (termed 45S5 Bioglass®) [18] has a very slow degradation rate because it converts to a HAp-like material in the physiologic environment [96, 97]. Typically, bioceramics degrade via multiple mechanisms: physiochemical dissolution accompanied by possible phase transformation, multinucleated cell-mediated degradation, and mechanical fragmentation due to loss of structural integrity by the two former mechanisms [76].

Although bioceramics have inviting qualities, they are extremely brittle and difficult to shape into the desired structures because of their stiffness and low flexibility and moldability [98]. They have weak mechanical strength [99] and fracture toughness [100], which limit their applications to non-load-bearing areas. However, their combination with mechanically strong biomaterials, such as synthetic polyesters or metals, tends to eliminate brittleness, difficulty in shaping, and weak mechanical strength [101, 102].

### 3.4. Metals

Metallic biomaterials are extensively applied in dental and orthopaedic fields to support the replacement of lost bone structures because of their excellent mechanical properties [103, 104]; they display high strength, toughness, and hardness, in comparison to polymers and ceramics, making them suitable for applications in load-bearing areas [105]. It is reported that metals enhance the mechanical properties of a scaffold by decreasing the pore size [106].

Within this group of biomaterials, titanium and titanium alloys are encouraged in bone regeneration due to their high biocompatibility, appropriate mechanical properties, and elasticity [107]. Different studies reported that titanium-based 3D scaffolds display good hydrophilicity, which enhances mineral deposition and encourages cell attachment and proliferation* in vitro* [107] and new bone formation without any signs of inflammation or necrosis* in vivo* [108].

Nonetheless, lack of biodegradability of titanium and titanium alloys is a major disadvantage and might discourage their applications in bone regeneration due to the need of a second surgery for removal, which can compromise patient satisfaction and increase health care costs [103].

In the past decade, magnesium and magnesium alloys have been thoroughly researched and found to be extremely appealing materials for orthopaedic applications [103] with great potential in BTE; they have mechanical properties close to native bone and are completely biodegradable [103] which eliminates the need for a second surgery to retrieve the scaffold. Although magnesium and magnesium alloys degrade by corrosion [109], their byproducts are biocompatible and do not elicit adverse reactions that could negatively affect surrounding tissues [110].

Magnesium and its alloys are osteoconductive, play a role in cell attachment [103], and tend to increase the expression of osteogenic markers* in vitro* [111]. Although pure magnesium has a rapid rate of degradation* in vivo* [112], this can be controlled through the utilization of magnesium alloys [113] or by coating pure magnesium with titanium [114] or ceramics [115]. Similar to natural and synthetic polymers, metals lack* bioactivity*.

In regard to all the previously described biomaterials, each has remarkable characteristics and individual limitations. Henceforth, it is very common to combine two or more different biomaterials to produce a “*synergistic effect*” in the overall resulting properties [116] and improve the mechanical, biological, and degradation kinetics of a scaffold [117]. Additionally, bone tissue is made of organic and inorganic components [118], thereby making it more difficult for one biomaterial to simulate the complex bone tissue environment and possess the required characteristics of the target tissue [21]. These scaffolds are referred to as “composite” or “hybrid” and whenever three biomaterials are incorporated the term “ternary” can be used. Composite scaffolds used for BTE applications are divided into “*polymer/ceramic*,” “*ceramic/metal*,” and “*polymer/metal*.” The former type is the most popular among composites and has been thoroughly studied by researchers in the orthopaedic field for the last five years [119]. However, the literature confirms that various composite scaffolds support attachment, proliferation, and differentiation of osteoblasts while maintaining the final shape of newly formed bone [119].

Composites, whether ternary or not, consist of a major component (matrix) and minor components (filler); the material which constitutes more than 50% of the blend is considered the major element, while the material/materials that are less than 50% represent the minor component [120].

## 4. Advances in 3D Scaffold Fabrication Techniques

Different techniques are employed in the fabrication of 3D scaffolds, with the conventional methods including particle leaching, gas foaming, freeze drying, phase separation, fiber meshes/fiber bonding, melt molding, and solution casting [14]. However, heterogeneities in pore size, porosity, interconnectivity, and architecture are unavoidable with these techniques, which can complicate drawing conclusions from experiments that assess the effect of scaffold properties on newly formed tissues [121]. Moreover, these techniques might not be applicable for the fabrication of a custom-made scaffold with finely tuned architecture that replicates the complexity of native tissues and precisely conforms to the shape of a certain defect.

With the development of solid-freeform fabrication (SFF) techniques, also known as rapid prototyping (RP), it became possible to create scaffolds with precise external shape, internal morphology, and “reproducible” three-dimensional architecture, despite their complexity [122].

**Figure 2:**
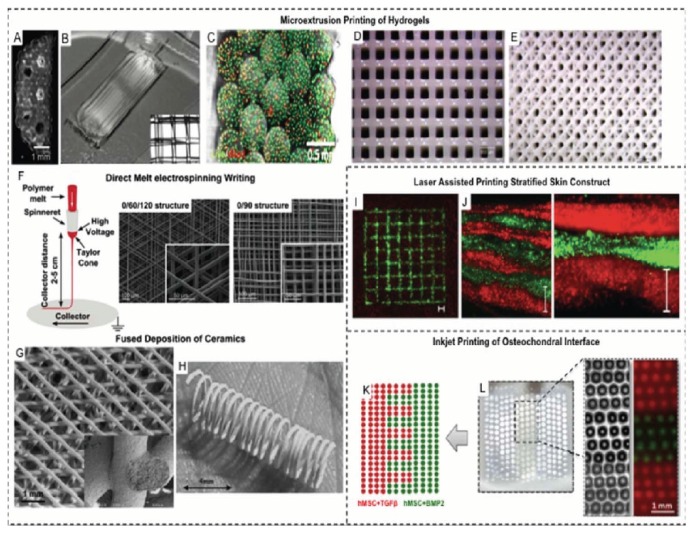
Different 3D printing methods used to manufacture 3D scaffolds for various applications. Courtesy of Obregon et al., 2015 [123].

These technologies represent additive manufacturing as they build complex structures layer by layer by “3D printing,” with one of the following techniques: inkjet printing, laser-assisted printing (e.g., Selective Laser Sintering (SLS) and Stereolithography (SLA)), and extrusion printing (e.g., fused deposition modeling (FDM)) [123]. Each printing method is compatible with specific biomaterials and differs in resolution. For example, laser-assisted methods enable printing of diverse biomaterials with wide range viscosities [124]. Such diversity overcomes the limitations of inkjet printing in which low-viscosity inks are needed to prevent clogging of the nozzle of the printing machine that would eventually compromise printing quality, while extrusion printing is restricted to thermoplastic biomaterials such as PCL [123, 125]. In regard to bioprinting, inkjet, laser-assisted, and extrusion-based techniques are utilized in printing of living cells and constructs [123]. As a consequence, these technologies can be further explored in cell encapsulation and cell-based therapies, especially that they allow for controlled positioning of cells with precision, which could mimic the tissue interface and the surrounding microenvironment. However, these applications are generally reserved to hydrogel scaffolds [126], made of natural or synthetic polymers [125]. Different 3D printing methods are demonstrated in Figure 2 [123].

**Figure 3:**
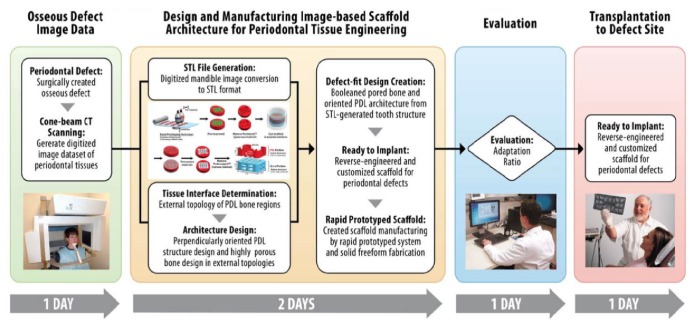
CAD models are produced based on computed tomography (CT) scans of a patient-specific bone defect to develop a custom-made bone graft substitute. Courtesy of Park et al., 2014 [128].

These new techniques utilize computer-aided design (CAD) and computer-assisted manufacturing (CAM) technologies to 3D-print a desired structure based on a CAD file that has already defined the exact dimensions of a scaffold [126]. This approach can be applicable in fabricating constructs that conform to a specific anatomical shape; in a typical clinical case scenario, CAD models are produced based on images from computed tomography (CT) scans of a patient-specific bone defect to develop a “custom-made” bone graft substitute which could be helpful in regenerating defects with complex geometry [127] as illustrated in Figure 3 [128]. Image-based 3D-printed scaffold following this scheme displayed promising results in preclinical investigations in periodontal regeneration with the need of further assessment for future employment in clinical practice [46, 128]. In the literature, few studies have focused on the concept of custom-made scaffolds for alveolar bone regeneration, by using subtractive technology (milling of a commercially available block, dictated by CAD/CAM technologies), which might not be as sophisticated due to the lack of layer-by-layer addition [129–131].

Although RP techniques are capable of producing constructs with satisfying mechanical strength by precisely controlling the overall geometrical design and porosity, these characteristics can still be limited by the machine's resolution and material repertoire. Due to the lack of sufficient resolution to fabricate nano- and submicrometer structures, a combination of RP techniques with different fabrication methods such as electrospinning [132] has been proposed to allow for the construction of efficient biomimetic constructs.

## 5. Applications of 3D-Printed and/or Compartmentalized Scaffolds in Alveolar Bone and Periodontal Tissue Regeneration

With the increased need for “optimal” tissue regeneration, “3D-printed” scaffolds have been recently investigated in different periodontal applications: guided bone regeneration (GBR), guided tissue regeneration (GTR), vertical bone augmentation, sinus augmentation, and socket preservation, showing variable outcomes of success.

PCL has been the most utilized biomaterial in these applications, probably because of its well-documented positive outcomes in hard tissue regeneration in the field of orthopaedics [119].

Regarding periodontal tissue regeneration, a novel anatomically shaped human-molar and rat-incisor 3D-printed PCL/HAp scaffold showed promising results in terms of inducing regeneration by “cell homing” instead of cell delivery in a rat model [133]. In another rat model [46, 128], the concept of “compartmentalization” was applied to achieve regeneration of periodontal ligament, cementum, and alveolar bone, by utilizing a custom-made 3D-printed PCL scaffold which enclosed an alveolar bone interface and a PDL interface with fiber-guiding architecture. The biphasic construct allowed not only for the regeneration of obliquely oriented periodontal fibers, cementum-like tissue, and alveolar bone, but also for a greater control of tissue infiltration when compared to random porous scaffolds. Similarly, multiphasic periodontal tissue regeneration was achieved with a 3D-printed PCL/HAp triphasic scaffold that allowed for spatiotemporal delivery of multiple proteins,* in vivo* [134].

Recently, a biphasic PCL scaffold utilizing two scaffold fabrication techniques and cell sheet technology was investigated in the regeneration of the alveolar bone and periodontal tissues [33]. In fact, cell sheet technology was tested as a part of the scaffold to provide biomechanical support during wound healing process, which was lacking in a material-free approach of cell sheet technology in periodontal regeneration [135]. The scaffold enclosed two compartments manufactured by two different techniques and of different biomaterials: the bone compartment was constructed from *β*-TCP/PCL by fused deposition modeling (FDM) and then thermally incorporated with an electrospun PCL membrane enclosing cell sheets, representing the PDL compartment. After being tested in a subcutaneous rat model, results demonstrated successful regeneration of cementum, alveolar bone, and periodontal ligament. Early bone markers confirmed that FDM bone interface promoted early bone formation. However, there was no functional orientation of the PDL fibers, as no specific cell oriented architecture was contained in the design. To address this finding, the researchers developed a second generation of the same scaffold [34] but with specific modifications of the PDL compartment, by including superimposed concentrically oriented rings in the membrane, fabricated by melt electrospinning to allow for some level of tissue organization. This interface was also more porous to improve cell interactions and vascularization. The bone compartment was modified to enhance alveolar bone regeneration by coating the *β*-TCP/PCL construct with calcium phosphate (CaP). By employing the same animal model, results revealed higher bone formation with improved PDL fiber orientation and vessel ingrowth.

Despite the promising results* in vivo*, 3D-printed PCL-based scaffolds showed less promising outcomes in clinical studies.

**Figure 4:**
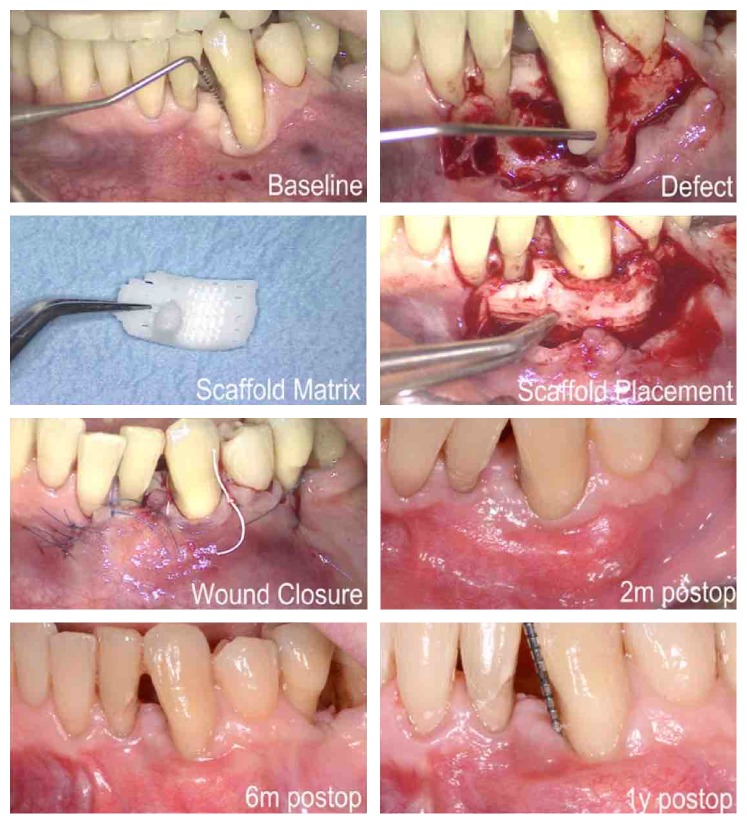
Custom-made 3D-printed PCL/HAp scaffold based on images from computed tomography (CT) scans and combined with CAD/CAM technologies for periodontal tissue regeneration. Courtesy of Rasperini et al., 2015 [136].

A “*prefabricated*” 3D PCL scaffold printed by FDM was tested for socket preservation in a randomized clinical trial [35]. Although the scaffold maintained the ridge height better after 6 months, this finding can be expected because no filler was used in the control group. The efficacy of PCL-based scaffolds as space fillers in socket preservation should be interpreted with caution, because comparison with other socket preservation techniques is still lacking. Most importantly, the scaffolds showed minimal signs of degradation 6 months following intervention and fibrous invasion was reported in one patient due to manual shaping for friction fit within the extraction socket. One might conclude that “*custom-made*” 3D-printed PCL scaffolds based on medical imaging could show more favorable results by allowing precise adaptation to the bony defect. However, adverse outcomes were reported when a custom-made image-based 3D fiber-guiding PCL/HAp scaffold printed by SLS was applied in GTR in a recent case report, as shown in Figure 4 [136]. After thirteen months of scaffold implantation, soft tissue dehiscence was reported with histological and molecular weight analysis revealing that almost 76% of the scaffold mass remained with minimal bone repair. This result can be interpreted by the very slow degradation profile of PCL in addition to its inferior cell affinity and weak osteoconductive activity. The final outcome might have also been compromised due to the low resolution of the applied 3D printing technology. Interestingly, one might attribute the end result to the acidic byproducts upon degradation, as well. Nonetheless, this matter is debatable, as some data in the literature have revealed that metabolic pathways easily remove PCL byproducts and thus PCL does not produce a local acidic environment as other aliphatic polyesters [137, 138].

The slow degradation of PCL has been considered appealing in hard tissue regeneration [40], but this might be valid for orthopaedic applications only, because there are key differences in the behaviour between long bones and alveolar bone as remodeling is slower in the former in comparison to the latter [139]. Although it is very well documented that bioceramics tend to control the degradation rate of polyesters [140], the percentage of HAp that was combined with PCL in this case report (4%) might not have been sufficient to accelerate the degradation profile. As a matter of fact, accelerated degradation of PCL was achievable with a much higher percentage of HAp in an* in vitro* investigation [140].

Interestingly, this fiber-guiding scaffold model in GTR was successful in preclinical studies on rats [46, 128]. The discrepancy in results could be due to the differences between rats and humans in terms of healing window, anatomic structures, and host responses [141].

Another biomaterial that has been widely tested as part of 3D scaffolds for periodontal applications is bioceramics, mainly in sinus and bone augmentation procedures. In a sheep animal model, a prefabricated 3D-printed scaffold, made of biphasic ceramic (*α*-TCP + HAp), was compared to bovine bone (Bio-Oss) and particulate *β*-TCP for vertical bone augmentation [142]. The scaffold eliminated the need for membranes and provided better mechanical support to the newly formed tissues, which can be explained by the fact that when *α*-TCP comes into contact with body fluids, it converts to HAp which has a very slow degradation rate. Similarly, a 3D-printed BCP scaffold (HAp (30%), *β*-TCP (60%), and *α*-TCP (10%)) showed favorable outcomes as a bone graft substitute for sinus augmentation* in vivo* in terms of abundant deposition of newly formed bone tissue within the biomaterial pores, which could be promising in future clinical applications [143].

Specific conclusions can be extrapolated from the previous studies about the use of certain biomaterials in scaffolding for various periodontal applications. For example, the use of PCL as the only biomaterial in a scaffold could be discouraged mainly due to its slow degradation rate which can lead to wound dehiscence and subsequent failure of tissue regeneration, also due to its inferior cell affinity [71]. If combined with bioceramics, an increase in the weight percentage of the bioceramic should be utilized to accelerate the degradation profile. Likewise, increased porosity of the bulk scaffold construct can assist with more rapid tissue ingrowth that can further drive the degradation process. Other aliphatic polyesters might be discouraged as well due to their acidic byproducts unless counteracted by the combination of bioceramics or metals. In a recent* in vitro/in vivo* investigation, magnesium/PLGA scaffold was applied in socket preservation, in which magnesium was able to counteract the acidic degradation of PLGA, thus decreasing the risk for tissue inflammation and eventually enhancing osteogenesis [79]. Still, it should be kept in mind that the ideal percentage of biomaterials to eliminate the risk of adverse effects may be difficult to determine for clinical uses.

Regarding GTR, where contact with bacteria and exposure are more likely to occur, natural polymers could be the best choice for this specific application, such as chitosan which has antibacterial properties that could decrease the chance of bacterial contamination and subsequent exposure. Gelatin can also be recommended in this application, and it has already been investigated* in vitro* as the biomaterial of a “periodontally inspired” scaffold, created by directional freeze-casting [144]. Despite having relatively low compressive resistance, gelatin displayed attractive biological properties because intrinsic cell interactions with the scaffold surface are still possible in the presence of adhesive RGD motifs, making cell affinity and growth more significant [145]. To overcome the mechanical weakness of gelatin, incorporation of this platform into the previously described synthetic polymer-based, fiber-guiding 3D scaffold system [46, 128] has been proposed.

It must be taken into consideration that natural polymers must be combined with mechanically strong materials; in GTR applications, the scaffold serves a dual role: a grafting material and a membrane. Since space maintenance is required for periodontal regeneration, it is essential to utilize a mechanically strong scaffold.

For applications in alveolar bone regeneration, augmentation, and socket preservation, scaffolds made of bioceramics can be recommended. Nevertheless, using bioceramics alone can be questionable for clinical applications, because of their weak mechanical properties. To overcome such limitations, bioceramics can be combined with mechanically strong biomaterials as mentioned earlier.

In non-load-bearing areas, collagen could be the preferred biomaterial in such combination. Better outcomes are to be expected with the incorporation of collagen because a bioceramic/collagen mix is the closest replicate of the ECM composition of native bone [146].

Specifically, the combination of collagen with* hydroxyapatite* is encouraged in bone tissue regeneration [146] due to the compositional similarities to native tissue and reasonable degradation rates for clinical uses [90].

In bone tissue regeneration, care must be taken that this process might take a long time in case of severe ridge resorption, because bone regeneration through scaffolds commences at the peripheries, where contact points between the biomaterial and native bone exist. However, this can be resolved with advances in tissue engineering and further investigations, by creating different points of bone nucleation through engineering with stem/osteoprogenitor cells [143].

To this end, studies on 3D-printed scaffolds in the periodontal field have focused on biomaterials, new and/or functional tissue formation, and spatial organization mainly when multiple tissue regeneration was attempted. Accordingly, other characteristics still need to be addressed more thoroughly, such as vascularization, analysis of landscape topography, and degradation profile and kinetics. Moreover, “*image-based*” 3D-printed scaffolds must be investigated in alveolar bone regeneration prior to placement of dental implants, as there are no published studies on this specific use.

## 6. Recommendations and Future Directions

BTE is based not only on cellular and molecular events and interactions, but also on the development of biomaterials and scaffolds with prescribed biomechanical properties, representing a fundamental part of the BTE paradigm.

Dental literature on 3D scaffolds and related biomaterials as alternative to bone grafts is still scarce, with extremely limited clinical trials. Validation of the efficacy of scaffolds tested in animal models is obligatory, because the already published results are not representative due to small defects, graft size, and also a completely different healing process in small animals. Randomized controlled clinical trials are mandatory, with adequate number of patients and long-term follow-up of implant therapy following scaffold employment in preimplant augmentation procedures. Thorough evaluation of biological and mechanical properties, as well as degradation profiles of 3D scaffolds in periodontal applications, is needed. The effect of 3D scaffolds on “blood clot stabilization” should be assessed, as it is an important prognostic factor in alveolar bone regeneration [7]. Moreover, scaffolds should be tested as part of a complete tissue regeneration protocol, in combination with new techniques of soft tissue management which is the key for optimum regenerative outcomes [147]. Due to the existing limitations of scaffold fabrication techniques, investigations of technique combination must be evaluated as an acceptable modality for producing scaffolds with clear-cut scales on different levels. As scaffold stabilization represents an important factor in preventing micromotion and compromised regeneration outcomes, different stabilization techniques could be investigated as well (press-fit graft, fibrin glue) since fixation with screws and pins might compromise the scaffold integrity, especially in large defects.

## 7. Conclusions

Scaffolding matrices are an attractive alternative to bone replacement grafts in surgical procedures related to endosseous implant placement, that is, vertical and/or horizontal bone augmentation, socket preservation, and sinus augmentation. Scaffolding matrices can also be used as a membrane and grafting material in periodontal tissue regeneration. A scaffold should be biocompatible, biodegradable, and bioactive and should be made of a hybrid of biomaterials, as the combination of different biomaterials is superior to a pure material, mechanically and biologically. Regardless, it is still unknown which combination of materials is optimal for alveolar bone regeneration. Much work lies ahead to translate the promising results of preclinical studies into clinical reality.
